# Pu-erh Tea Ameliorates Atherosclerosis Associated with Promoting Macrophage Apoptosis by Reducing NF-*κ*B Activation in ApoE Knockout Mice

**DOI:** 10.1155/2018/3197829

**Published:** 2018-08-23

**Authors:** Yihui Xiao, Ming He, Xiao Liang, Jianqing She, Lan He, Yan Liu, Juan Zou, Zuyi Yuan

**Affiliations:** ^1^Department of Cardiovascular Medicine, First Affiliated Hospital of Xi'an Jiaotong University, 277 Yanta West Road, Xi'an, Shaanxi 710061, China; ^2^Department of Rheumatology, First Affiliated Hospital of Xi'an Jiaotong University, 277 Yanta West Road, Xi'an, Shaanxi 710061, China; ^3^Key Laboratory of Environment and Genes Related to Diseases, Xi'an Jiaotong University, Ministry of Education, Xi'an, Shaanxi 710061, China; ^4^Key Laboratory of Molecular Cardiology, Xi'an Jiaotong University, Xi'an, Shaanxi 710061, China

## Abstract

We explored whether pu-erh tea consumption ameliorates atherosclerosis and the possible mechanism for its effects in apolipoprotein E-deficient (ApoE^−/−^) mice. Our data showed that pu-erh tea consumption markedly reduced early fatty streak formation and the advanced fibrofatty plaque sizes. Additionally, the mean proportion of inflammatory macrophages in the plaque decreased, and the number of apoptotic macrophages increased significantly. NF-*κ*B activity in peritoneal macrophages decreased by 75.6% compared to the controls, similar with the levels of IL-6, IL-12, and TNF-*α* expression. The tea extract increased the apoptosis of RAW264.7 cells by decreasing NF-*κ*B activation and reducing the inflammatory cytokine expression. In conclusion, pu-erh tea ameliorates atherosclerosis progress by alleviating the chronic inflammatory state by reducing NF-*κ*B activation and promoting macrophage apoptosis in atherosclerotic plaques.

## 1. Introduction

In recent years, atherosclerosis has been considered a chronic inflammatory disease [1]. In cases of low-grade inflammation and under the effects of various cytokines, circulating monocytes continue to adhere to the vascular endothelium, roll and migrate into the arterial endothelium, convert into macrophages, and eventually to foam cells [2]. At the same time, macrophages secrete large amounts of proinflammatory cytokines, further recruiting and activating macrophages [3]. The number and function of macrophages in the plaque directly affect the size of the plaque and the deposition of lipids [4]. Previous studies have shown that macrophage apoptosis plays an important role in atherosclerosis progress, and when macrophage apoptosis decreased in the arterial plaques, the atherosclerosis accelerated [5, 6].

Fermented black tea is known to modulate blood lipids and provide anti-inflammatory effects and protection against antioxidative stress [7–10]. Pu-erh tea, a black tea produced in Yunnan, China, is a postfermented (secondary fermentation) tea made from the large sundried, raw, green Yunnan leaves. It has been suggested that pu-erh tea might be useful in the treatment of chronic inflammatory diseases, including atherosclerosis. However, no studies have reported if the habitual drinking of pu-erh tea has beneficial effects on atherosclerosis.

In this study, we used apolipoprotein E-deficient (ApoE^−/−^) mice to investigate if pu-erh tea drinking ameliorates atherosclerosis in early fatty streak formation and advanced fibrofatty plaque progression, focusing on anti-inflammatory effects and modulating macrophage apoptosis.

## 2. Methods

### 2.1. Experimental Atherosclerosis

ApoE^−/−^ mice (B6.129P2-ApoE^tm1Unc^) were kept in a temperature-controlled facility on a 12 h light-dark cycle with free access to food and water. After being weaned at 4 weeks of age, mice were fed a normal chow diet until 6 weeks of age, when the animals were switched to a western diet containing 21% fat and 0.15% cholesterol. ApoE null mice in the pu-erh tea group were fed pu-erh tea for 8 weeks or 16 weeks, whereas the mice in the control group were fed with ddH_2_O changed daily at 8 am. The animal investigation conformed to the Guide for the Care and Use of Laboratory Animals published by the United States National Institutes of Health (NIH Publication number 85–23, revised 1996). The protocols for mouse sacrifice and tissue harvest were approved by the Institutional Ethics Committee for Animal Experiments of Xi'an Jiaotong University. The mice were sacrificed after anaesthesia using 2% avertin intraperitoneally (0.25 mg/g body weight). Briefly, the blood was obtained from left ventricular puncture and the heart and thoracic aorta were removed under a microscope after perfusion by sterile phosphate-buffered saline (PBS). Macrophages were collected after peritoneal lavage by PBS, as previously described [11].

### 2.2. Preparation of Tea for Feeding or Cell Culture

The commercial pu-erh tea samples were collected from the Yunnan Province of China. Voucher specimens of tea *(Camellia sinensis* var. *assamica* (L.) *O. Kuntze*; Theaceae) were collected from agroforests and deposited at the Kunming Institute of Botany of the Chinese Academy of Sciences [12]. Previous studies have shown the detailed processing of pu-erh tea [8]. Three grams of pu-erh tea (The Top of Mini Gold Fermented Pu-erh Tea, Colorful Yunnan Qing Feng Xiang Tea Industry Co. Ltd., Kunming, China) was placed in a gauze bag, and then 500 ml boiling water was poured in with a dip for at least 10 minutes. Once it had cooled naturally, the tea was used for feeding the animals after filtration, sterilization, and subpackaging. The solid content was dislodged with intermediate speed quantitative filter paper, and all liquids were mixed. Ten milliliters of the liquids was diluted with sterile ddH_2_O after filtering with a 0.22 ìm aperture filter. Water extracts of different concentrations were used to treat cells, with 20 *μ*l of the tea/water extracts added to 1 ml medium. The content of biochemical ingredients, including the total polyphenols, total flavonoids, free amino acids, theaflavins, thearubigins, and theabrownins, in pu-erh tea was analysed in a previous study [13].

### 2.3. Detection of Lipids in the Serum

Serum was separated by centrifuging venous blood from mice at 3000 rpm for 15 minutes, and the total cholesterol (TC) and triglyceride (TG) levels were measured using assay kits (Dong'ou, Wenzhou, China) according to the manufacturer's instructions. Glucose was measured in peripheral blood with an ACCU-CHEK Advantage glucometer (Roche Diagnostics Inc., Penzberg, Germany). Each sample was tested three times.

### 2.4. Cell Culture

RAW264.7 macrophage cells were obtained from the Fourth Military Medical University (Xi'an, PR China) and were cultured in high-glucose DMEM medium containing 10% heat-inactivated foetal bovine serum (FBS), 100 U/ml penicillin, and 100 U/ml streptomycin. The RAW264.7 cells were plated at a density of 1 × 10^6^/ml maintained at 37°C in a humidified atmosphere containing 5% CO_2_. Cells were treated with different concentrations (1 : 10 added to medium) of pu-erh tea/water extract and different intervention times. Some group cells were pretreated with PMA (100 ng/ml) or BAY11-7085 (10 *μ*M) for 2 hours.

### 2.5. Chemical and Immunochemistry Staining

Oil Red O staining was used to detect lipids in the plaque, and the sections were analysed using polarization microscopy. Immunohistochemistry was performed with antibodies to identify CD68 (1 : 100 dilution, Serotec, Kidlington, UK) overnight. Negative controls in the absence of primary antibodies were also performed. The samples were then incubated with horseradish peroxidase-conjugated or fluorescein isothiocyanat-conjugated goat anti-mouse secondary antibodies (1 : 2000 dilution, Pierce, Rockford, USA) for one hour. The nuclei were stained with DAPI (Molecular Probes Inc., Eugene, OR). Section images were captured digitally using an Olympus BX51 imaging system (Olympus, Tokyo, Japan) and were quantified with Image-Pro Plus 6.0 software. The cross-sectional surface area of the lesion and total cross-sectional vessel area were also quantified.

### 2.6. Cell Apoptosis Assay

The In Situ Cell Death Detection Kit and TMR red (Roche, Mannheim, Germany) were used to detect cell apoptosis in cryopreserved tissue sections or 96-well microplates, according to the manufacturer's instructions. Briefly, 4% paraformaldehyde was used to fix the cell or tissue, and 0.1% Triton X-100 was used for permeability. The samples were rinsed twice with PBS and dried. Fifty microliters of TUNEL reaction mixture was added to each sample. One sample from each group was treated with the TUNEL reaction mixture without terminal transferase to act as a negative control. The slides were incubated in a humidified atmosphere for 60 min at 37°C in the dark. The samples were rinsed with PBS 3 times. The samples were then directly analysed under a fluorescence microscope.

### 2.7. RNA Extraction and Quantitative PCR

The total RNA was isolated from the aorta and peritoneal macrophages of mice, and RAW264.7 cells were isolated with TRIzol reagent (Invitrogen, Carlsbad, CA). The Nanodrop 1000 (Thermo, Wilmington, USA) was used to quantify the total RNA. The resulting RNA was reverse transcribed and analysed by quantitative PCR with SYBR PrimeScript™ RT-PCR Kit (Takara, Dalian, China). All real-time reactions were performed on the iQ5™ Multicolor Real-Time PCR detection system (Bio-Rad, Hercules, USA). A three-step PCR procedure of 5 s at 95°C, 20 s at 63.5°C, and 10 s at 72°C was applied for 45 cycles. GAPDH was used as a housekeeping gene. The primer sequences are shown in Table 1. The data were analysed using the 2^−ΔΔCT^ method.

### 2.8. Western Blot Analysis

Proteins from the peritoneal macrophages and RAW264.7 cells were extracted by RIPA buffer (Cybrdi Inc., Rockville, USA) according to the manufacturer's instructions, and a protease inhibitor cocktail (Roche Molecular Biochemicals, Mannheim, Germany) was added to all samples. The BCA protein assay reagent kit (Pierce, Rockford, USA) was used to quantify the total protein. Equal amounts of protein extracts were separated with a 10% SDS-PAGE gel and then transferred to a nitrocellulose membrane using a Bio-Rad transfer blotting system (Bio-Rad, Hercules, USA). Five percent skim milk was used to block nonspecific binding for 1 hour at room temperature with slow shaking. The blots were incubated overnight at 4°C with anti-p65 (1 : 1000 dilution, Cell Signaling Technology, Danvers, USA), anti-I*κ*B-*β* (1 : 1000 dilution, Cell Signaling Technology, Danvers, USA), or anti-GAPDH (1 : 1000 dilution, Epitomics, Burlingame, USA). A horseradish peroxidase-conjugated anti-goat (1 : 10000 dilution, Abcam, Cambridge, USA) or anti-rabbit secondary antibody (1 : 5000 dilution, Abcam, Cambridge, USA) and enhanced chemiluminescent substrate (Pierce, Rockford, USA) were used for detection.

### 2.9. Electrophoretic Mobility Shift Assay (EMSA)

Nuclear proteins were isolated using NE-PER nuclear and cytoplasmic extraction reagents (Pierce, Rockford, USA) according to the manufacturer's instructions. A protease inhibitor cocktail (Roche Molecular Biochemicals; Mannheim, Germany) was added to all of the samples, and the BCA protein assay reagent kit (Pierce; Rockford, USA) was used to quantify nuclear proteins. All samples were stored at −80°C. EMSA was performed using an Odyssey Infrared Imaging System with NF-*κ*B IRDye-labelled oligonucleotides (LICOR Inc., Lincoln, USA). The DNA-binding reaction used 10 mg of nuclear extract mixed with oligonucleotide and gel shift-binding buffer (Tris-HCl 100 mM, pH 7.5, KCl 100 mM, DTT 2.5 mM, 0.25% Tween-20, MgCl2 5 mM, 20% glycerol, EDTA 2.5 mM, and polydeoxyinosinic-polydeoxycytidylic acid 1 mg/ml). Reactions occurred at room temperature in the dark for 30 minutes. Two microliters of orange loading dye (LICOR Inc., Lincoln, USA) was added and sample-loaded on the prerun 6% polyacrylamide gels. NF-*κ*B p65 antibody (1 : 1000, Cell Signaling Technology, Danvers, USA) and unlabelled NF-*κ*B oligonucleotides were used to confirm the supershift and specificity of NF-*κ*B DNA-binding activity. The gels were scanned, and the signals were quantified using an Odyssey Infrared Imaging System and Odyssey software (LICOR Inc., Lincoln, USA).

### 2.10. RAW 264.7 Cell Migration Assay

Cultured RAW264.7 cells were treated with different concentrations of pu-erh tea/water extract for different durations, and a modified Boyden Chamber Assay (Transwell, Coster, Corning, Lowell, USA) was used to evaluate the migratory function of macrophages. Briefly, 5 × 10^4^ RAW264.7 cells were placed in the upper chambers of 24-well transwell plates with a polycarbonate membrane (8 *μ*m pores) that contained 300 *μ*l DMEM with 1% FBS; 800 *μ*l DMEM with 10% FBS was added to the lower chamber. LPS (10 ng/ml) was added to the medium and placed in the lower chambers. After incubation for 12 hours at 37°C, the membrane was washed briefly with PBS and fixed with 4% paraformaldehyde. The upper side of the membrane was wiped gently with a cotton ball. The membrane was then stained with a haematoxylin solution and carefully removed. The amount of RAW264.7 migration was evaluated by counting the migrated cells in six random high-power (×100) microscopic fields. Triplicate studies were performed for each experimental condition.

### 2.11. Cholesterol Efflux Assay

Macrophages were treated with various concentrations of pu-erh tea/water extract for different durations, followed by the equilibration of NBD cholesterol (1 *μ*g/ml) for an additional 6 hours, with or without ox-LDL (30 *μ*g/ml). The green NBD cholesterol-labelled cells were counted using a fluorescence imaging system (Olympus, Tokyo, Japan) and then washed with PBS and incubated in DMEM medium for 6 hours. The fluorescence-labelled cholesterol released from the cells into the medium was measured with a multilabel counter (Synergy HT Multi-Mode Microplate Reader, BioTek Instruments Inc., Winooski, USA). The cholesterol efflux was expressed as a percentage of fluorescence in the medium relative to the total amount of fluorescence (cells and medium).

### 2.12. Quantitative Measurement of Intracellular Cholesterol Content

The total cholesterol and free cholesterol were analysed using an enzymatic fluorometric method described previously by Heider. In brief, RAW264.7 cells were harvested and washed twice with PBS, and lipids were extracted by adding 500 *μ*l of isopropanol to the cell pellet. After sonification, the samples were centrifuged for 15 minutes at 800 ×g, and the clear supernatant was collected for total and free cholesterol analysis (Cayman Chemical, Ann Arbor, USA) and measured in a TECAN GENios Pro (excitation, 325 nm; emission, 415 nm). The concentration of total and free cholesterol was analysed using a standard curve and normalized by measuring the concentration of the total cell protein using the BCA protein assay. The cholesterol ester concentration was calculated using the total cholesterol minus the free cholesterol.

### 2.13. Statistics Analysis

Data were expressed as the means ± SD or indicated in the legends. Student's *t*-test or one-way ANOVA was performed to analyse the variance among groups. A value of *p* < 0.05 was considered to be statistically significant.

## 3. Results

### 3.1. Pu-erh Tea Consumption Reduced Early Fatty Streak Formation and Advanced Fibrofatty Plaque Progression in ApoE Null Mice, although No Effect Was Observed on the Physiological Parameters

**Figure 1:**
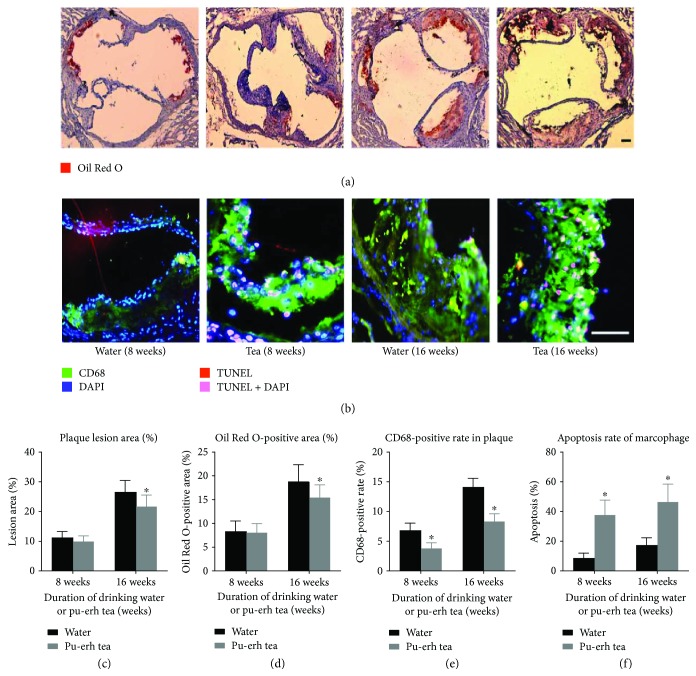
Pu-erh tea consumption could inhibit the progress of atherosclerosis by promoting macrophage apoptosis. (a-b) Oil Red O (ORO) staining to detect lipid deposition and triple immunofluorescence to detect the apoptosis of macrophages in the plaques, magnification ×40 (a) or ×100 (b), bar = 200 *μ*m. (c–f) Plaque lesion area, ORO staining positive rate, CD68 positive rate, and apoptosis rate of macrophages in the plaque of ApoE null mice. ^∗^*p* < 0.05.

Early lesions, as fatty streaks, were induced into the root of the aorta in all 8-week pu-erh tea consumption groups, and advanced fibrofatty plaques were formed in all 16-week pu-erh tea consumption groups. Compared to the controls, the lesion area fraction (fatty streaks) at the aortic root in the ApoE null mice in the 8-week pu-erh tea consumption group was reduced. Furthermore, the advanced fibrofatty plaque area fraction was reduced significantly in the ApoE knockout mice that were subjected to pu-erh tea consumption for 16 weeks (*p* < 0.05). Additionally, the mean ratios of Oil Red O-positive regions were also reduced for 8 weeks, and these were reduced significantly for 16 weeks (*p* < 0.05). (Figures 1(a), 1(c), and 1(d)).

After consuming pu-erh tea for either 8 weeks or 16 weeks, no significant difference was found in the blood cholesterol, triglyceride levels, and glucose in ApoE null mice compared to the controls.

### 3.2. Pu-erh Tea Consumption Reduced the Proportion of Inflammatory Macrophages and Increased Macrophage Apoptosis in the Plaques of ApoE Null Mice

The mean proportion of macrophages in the plaques of ApoE null mice that consumed pu-erh tea for 8 and 16 weeks was reduced by 45.2% and 41.3%, respectively, compared to the control (both *p* < 0.05). After 8 weeks of pu-erh tea consumption, the mean ratio of the apoptotic macrophages to the total macrophages in the plaque was increased (37.4% versus 8.5% *p* < 0.01) compared to the control group. After 16 weeks of pu-erh tea consumption, the mean ratio of the apoptotic macrophages to the total macrophages in the plaque was increased (46.2% versus 17.1% *p* < 0.05) compared to the control group (Figures 1(b), 1(e), and 1(f)).

### 3.3. Pu-erh Tea Water Extract Increased Macrophage Apoptosis In Vitro

**Figure 2:**
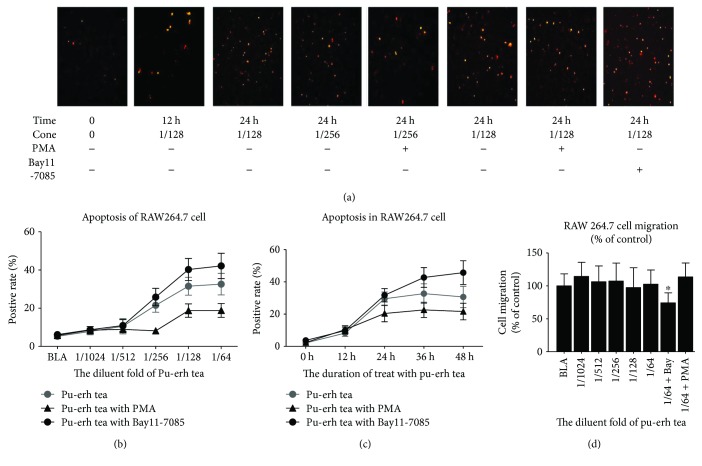
Apoptosis and migration of RAW264.7 cells treated with pu-erh tea/water extract. (a–c) Apoptosis rate of the RAW264.7 cells; cells in the same group were pretreated with PMA (5 *μ*M) or BAY11-7085 (10 mM) for 2 hours. (d) Migration of RAW264.7 cells. Magnification ×40, the bars represent the SEM. ^∗^*p* < 0.05.

RAW264.7 macrophages were treated with a certain concentration of pu-erh tea water extract, and the proportion of apoptotic cells was detected using the TUNEL technique. The data show that the proportion of apoptotic cells increased significantly in a time-dependent and dose-dependent manner (Figure 2).

### 3.4. Pu-erh Tea Consumption Reduced the NF-*κ*B Expression and Inhibited the DNA-Binding Activity of NF-*κ*B Both In Vivo and In Vitro

**Figure 3:**
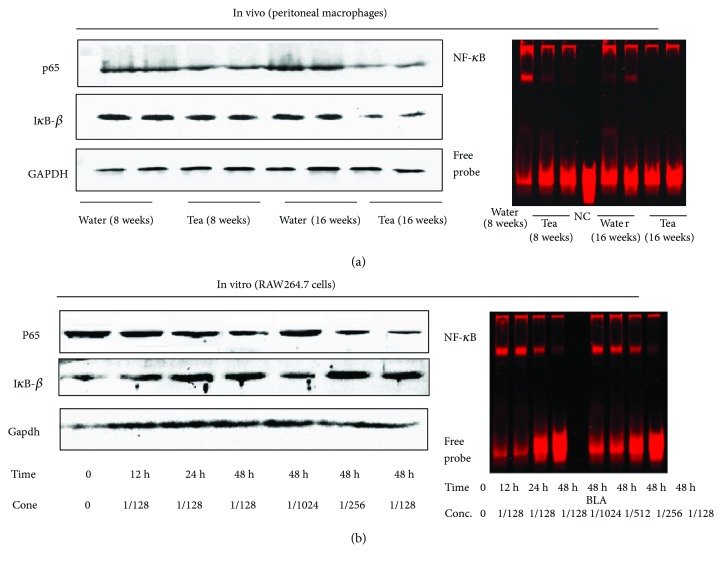
The effects of pu-erh tea consumption on the NF-*κ*B in vivo and in vitro. (a) Protein expression of p65, I*κ*B-*β*, and EMSA analysis of the NF-*κ*B-binding activity in peritoneal macrophages of ApoE null mice. (b) Protein expression of p65, I*κ*B-*β*, and EMSA analysis of NF-*κ*B-binding activity in RAW264.7 cells. GAPDH was detected as an internal reference.

**Figure 4:**
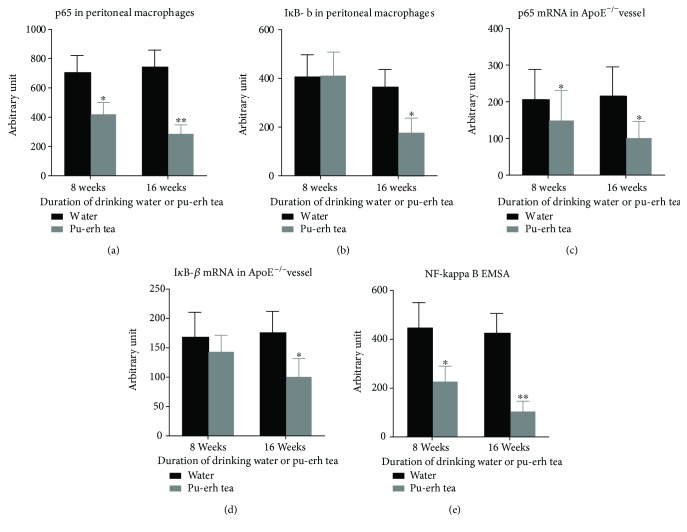
Quantitative analysis of pu-erh tea consumption on the protein and mRNA expression of p65, I*κ*B-*β*, and EMSA analysis of NF-*κ*B-binding activity in aortic tissue and peritoneal macrophages of ApoE null mice. (a–e) ^∗^*p* < 0.05 and ^∗∗^*p* < 0.01.

After drinking pu-erh tea for 8 and 16 weeks, the protein expression of p65 in peritoneal macrophages of ApoE null mice was reduced by 40.8% (*p* < 0.05) and 61.8% (*p* < 0.01), respectively. The expression of I*κ*B-*β* was reduced slightly in the 8-week treatment group and markedly by 52.3% (*p* < 0.05) in the 16-week treatment group. The p65 mRNA expression that RNA extracted from the aortic tissue of ApoE null mice was decreased by 28.1% and 53.6%, respectively (both *p* < 0.05), and the I*κ*B-*β* mRNA expression were both reduced, but more significantly in the 16-week treatment group (*p* < 0.05). EMSA was performed to determine the DNA-binding activity of NF-*κ*B in the nuclear proteins extracted from mouse peritoneal macrophages. The mean grey values of the binding activity of NF-*κ*B were decreased by 49.4% (*p* < 0.05) and 75.6% (*p* < 0.01), respectively (Figures 3 and 4).

**Figure 5:**
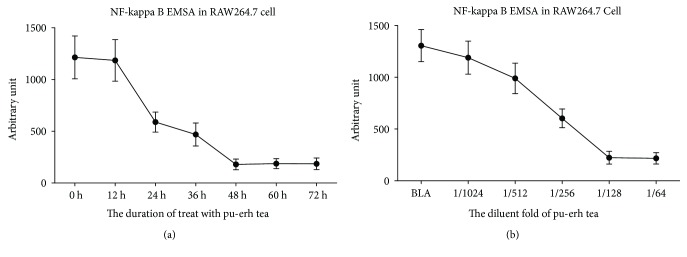
Quantitative analysis of EMSA for the assessment of the NF-*κ*B-binding activity of RAW264.7 cells; the bars represent the SEM.

The RAW264.7 cells were treated with pu-erh tea water extract, and the expression levels of the p65 protein and mRNA in cells were reduced markedly. In contrast, the I*κ*B-*β* expression in both the protein and mRNA levels was increased significantly. The effects of pu-erh tea water extract on the p65 and I*κ*B-*β* protein and mRNA expression levels were in a dose-dependent and time-dependent manner. The binding activity of NF-*κ*B by EMSA was also decreased in a dose-dependent and time-dependent manner (Figure 5).

### 3.5. Pu-erh Tea Consumption Decreased Inflammatory Cytokine Expression in the Vascular Tissue of ApoE Knockout Mice and in RAW264.7 Cells

**Figure 6:**
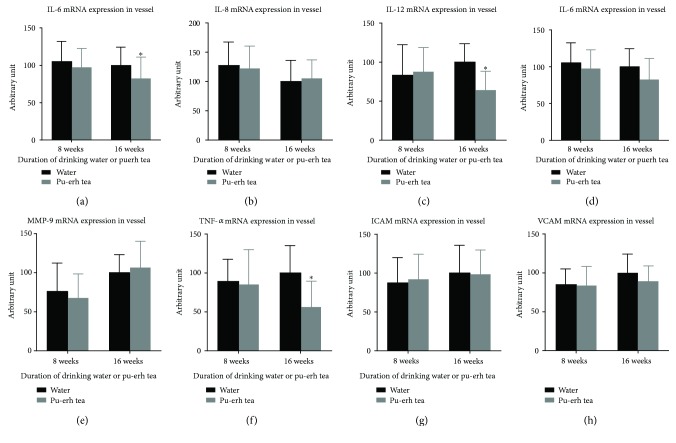
The effects of pu-erh tea consumption on the expression of various cytokines in the aortic vascular tissue of ApoE null mice. (a–e) ^∗^*p* < 0.05.

After ApoE null mice drank pu-erh tea for 16 weeks, the relative mRNA expression levels of IL-6, IL-12, and TNF-*α* in their aortic tissue decreased by 17.83%, 36.39%, and 43.73%, respectively, compared with those in the control groups (all, *p* < 0.05). No significant difference was discovered in its effect on the mRNA expression levels of IL-8, IL-18, MMP9, ICAM, and VCAM in vascular tissues between the 16-week consumption of pu-erh tea and those in the control groups (Figure 6).

RAW264.7 macrophages were treated with a certain concentration of pu-erh tea water extract, and the levels of IL-6, IL-12, and TNF-*α* were decreased significantly in a time-dependent and dose-dependent manner.

## 4. Discussion

In previous years, studies on the potential health beneficial effects of pu-erh tea have identified a range of biological activities, including antioxidation, antiobesity, anti-inflammation, anti-immunosenescence, antihyperlipidaemia, antitumor, antiviral, and antibacterial effects [14–20]. Clinical trials have suggested that tea consumption prevents the development of cardiovascular disease by a variety of mechanisms. For example, long-term tea drinking prevents coronary atherosclerosis [21]. In previous studies, each single component was extracted from the tea to assess the efficacy [22–24]. For example, the total polyphenols, main content of pu-erh tea, have the cardiovascular protection by various mechanisms, including anti-inflammatory properties, antioxidant capacities, improvement in endothelial function, inhibition of platelet aggregation, and antithrombotic properties [25]. Another content, flavonoids, the most important mechanism against cardiovascular diseases is their capacity to act as antioxidants [26, 27]. However, the effects of regularly consuming tea as a whole on atherosclerosis have not been studied in animals or humans. Our study indicated that 16 weeks of consumption of pu-erh tea reduced the plaque area and the lipid deposition at the aortic root of ApoE null mice. Our results provide the first demonstration of pu-erh tea's comprehensive role in the initiation and development of atherosclerosis.

Macrophages, derived from circulating monocytes, are highly active and mobile cells that function at multiple levels within the innate immune system. Macrophages are now recognized as key pathophysiologic agents in both early atherogenesis and advanced atherosclerotic plaque progression [28, 29]. During the initiation and development of atherosclerosis, abnormal endothelial cells secrete chemoattractants that lead to the recruitment of macrophages [30]. Thus, the prevention of monocyte entry or the induction of invaded macrophage apoptosis may have therapeutic effects on preventing or retarding atherogenesis. In this study, we observed that after 16 weeks of consumption of pu-erh tea, the number of macrophages in plaques was significantly reduced. However, the ox-LDL-induced macrophage foam cell formation and cholesterol efflux from the macrophages were not changed. Further studies reveal that pu-erh tea contributed to the apoptosis of macrophages in the atherosclerotic plaque, and this was further verified by our in vitro experiments. Additionally, we also found that the consumption of pu-erh tea did not change the adhesion and migration ability of macrophages. We thus conclude that pu-erh tea reduces the number of macrophages in the plaque by increasing the macrophage apoptosis rate. As mentioned above, pu-erh tea is comprised of a variety of biochemical ingredients. Polyphenols, the main contents of pu-erh tea, were reported to induce the apoptosis of macrophages in both macrophage cell line and in macrophages isolated from atherosclerotic ApoE null mice [31]. Therefore, we assume that the function of pu-erh tea on atherosclerosis is mainly dependent on polyphenols, but the mechanism has not been examined yet.

Park et al. found that NF-*κ*B activation inhibited the apoptosis of macrophages [32], which may relate to further cascade amplification reactions of inflammation. To clarify the possible mechanism that pu-erh tea increases the macrophage apoptosis rate, we made some work with the NF-*κ*B signaling. The transcription factor NF-*κ*B is one of the crucial regulators of inflammation [33]. NF-*κ*B belongs to a family of transcription factors consisting of five structurally related members: p65 (REL-A), REL, REL-B, p50, and p52. Upon stimulation, the IKK complex phosphorylates I*κ*B and triggers its ubiquitin-dependent degradation, resulting in the rapid and transient nuclear translocation NF-*κ*B, the transcription of downstream genes [34]. It was believed that NF-*κ*B signaling plays an important role in atherosclerosis development by controlling the transcription of many proinflammatory genes involved in atherosclerosis [35]. To verify the relationship between NF-*κ*B activation and macrophages, we observed that after drinking pu-erh tea, NF-*κ*B activity was decreased, and the proportion of inflammatory macrophages reduced because of the increase of apoptotic macrophages. By regulating the NF-*κ*B activity by PMA and BAY11-7085 in vitro, we proved that the pu-erh tea-induced macrophage apoptosis was mediated via the inhibition of the NF-*κ*B system. In vivo studies have shown that after 16 weeks of pu-erh tea consumption, the mRNA expression of tumor necrosis factor *α* (TNF-*α*), interleukin-6 (IL-6), and interleukin-12 (IL-12) in mouse arteries significantly decreased. The results from Li and Lin showed that NF-*κ*B activation may be mediated via TNF-*α* and its receptor [36]. With this result and the reduction in the activity of the NF-*κ*B system in our study, we can assume that the decreased TNF-*α* expression may result in decreased NF-*κ*B system activity, whereas the reduction of IL-6 and IL-12 may result from the reduction of the NF-*κ*B system.

Notably, there is some data different from previous study. In 1986, the Japanese scholar Sano used rats with a high-fat diet and the experiments found that Yunnan pu-erh tea lowered the serum total cholesterol and triglyceride levels [37]. Later studies concluded that drinking pu-erh tea played a role in reducing the circulating cholesterol and triglyceride levels as well as an antiatherosclerosis role [38]. Our results were inconsistent with those of the previous studies, possibly because of the different animal models we used. ApoE null mice are classic animal models used for studying atherosclerosis, in which the mechanism for arterial plaque formation came from spontaneous hypercholesterolemia. As a result, severe cholesterol metabolism disorders in this animal model may mask pu-erh tea's possible lipid-lowering effects found in other animal models. This result demonstrates that the decline of the arterial plaque area is not achieved through the regulation of blood lipids. Here, we consider that pu-erh tea inhibits the progress of atherosclerosis by alleviating the chronic inflammatory state rather than by regulating blood lipids in this model system.

## 5. Conclusion

Pu-erh tea is considered effective in regulating blood lipids and preventing atherosclerosis. Our research showed that in ApoE null mice, pu-erh tea impeded the progress of atherosclerosis by promoting the apoptosis of macrophages in the atherosclerotic plaque. However, whether the effect existed in humans remains uncertain, further work should be done.

## Figures and Tables

**Table 1:** The primer sequences of target genes.

Gene	Forward primer 5′–3′	Reverse primer 5′–3′
GAPDH	TCAACGGCACAGTCAAGG	ACTCCACGACATACTCAGC
IL-6	AGCCAGAGTCCTTCAGAGAGATAC	GCTAAGGACCAAGACCATCCAATT
TNF-*α*	GCTCTTCTGTCTACTGAACTTCGG	CCAGACCCTCACACTCAGATCAT
IL-12	ATGGCCATGTGGGAGCTGGAGAAAG	GTGGAGCAGCAGATGTGAGTGGCT
IL-8	CATAGCCATGTGGTTACTATCA	AGTAGCATGATCTTGAGAAGT
IL-18	CTCCCCACCTAACTTTGATG	CCAGGAACAATGGCTGCCAT
ICAM	GGAGACGCAGAGGACCTTAACAG	CGACGCCGCTCAGAAGAACC
VCAM	AATGCCATCCTCACCTTAATTGC	ATTCCACTTCTGCTTTGTCTCTCC
p65	GGATGGCTACTATGAGGCTGACC	GTCTGGATTCGCTGGCTAATGG
I*κ*B-*β*	CTGTGCCCGTGCCCTGCTTC	TCTGGGTTGGGTTGGGAATCC
MMP9	CCTGTGTGTTCCCGTTCATCTTTG	ATCCTGGTCATAGTTGGCTGTGG
ABCA1	CGCTTCGTCTCTCCGCTCTC	CCGCCTCACATCCTCATCCTC
ABCG1	TGTGCTGTTCGCTGCTCTGG	GGTAGGCTGGGATGGTGTCAAAG
CD36	GCGACATGATTAATGGCACAGAC	CCGAACACAGCGTAGATAGACC

## Data Availability

The data used to support the findings of this study are available from the corresponding author upon request.
